# Salvage of Thumb With a Free Skin Flap From the Great Toe

**Published:** 2018-08-13

**Authors:** Xingchen Li, Stephen Viviano, Ramazi Datiashvili

**Affiliations:** Division of Plastic Surgery, Department of Surgery, Rutgers University/New Jersey Medical School, Newark

**Keywords:** thumb reconstruction, hand surgery, microsurgery, free tissue transfer, salvage

## DESCRIPTION

**Figure 1:**
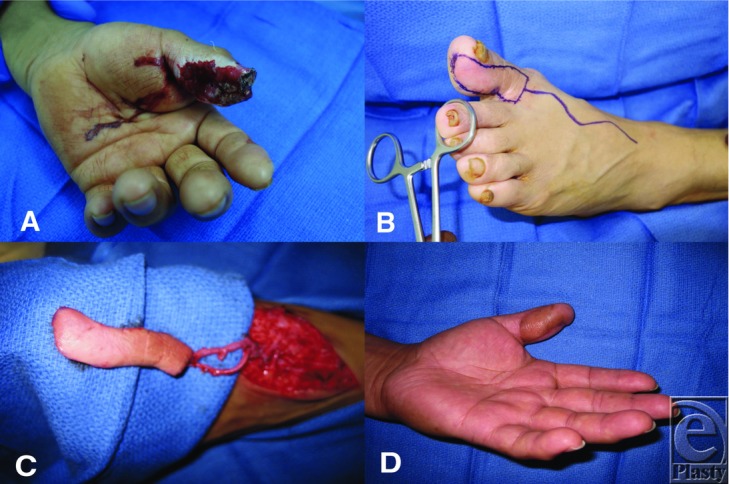
(a) Left thumb distal tip avulsion amputation with an extended full-thickness volar skin loss. (b) Intraoperative markings showing the lazy S-incision over the dorsalis pedis artery and the templated area of harvest. (c) Intraoperative view of the raised lateral toe flap with intact pedicle. (d) View of the left thumb 5 weeks after reconstruction.

The patient was a 52-year-old man who suffered an avulsion amputation injury to his left thumb. Examination revealed a left thumb distal tip amputation and a 7×3-cm open wound over the entire pulp and half of the proximal phalanx (Fig 1).

## QUESTIONS

1. What factors must be considered when approaching reconstruction of a volar thumb injury?
2. What local flap options exist for volar thumb reconstruction and what are the limitations?
3. What free flap options exist for volar thumb reconstruction?
4. What evidence exists for the use of lateral great toe flaps in the reconstruction of volar digital defects?

## DISCUSSION

### Background

Successful reconstruction of a thumb injury is crucial, as the thumb contributes to approximately 40% of the hand function.^1^ With any injury to the thumb volar aspect, the primary goals of reconstruction include reestablishment of durable, well-vascularized soft tissue and recovery of sensation in addition to soft tissue coverage.^1^ While multiple options exist for replacing the volar thumb pad, several factors must be considered. Ideally, the deficient tissue is replaced with like tissue, with its unique compartmentalized structure critical to grip, sensory, and proprioceptive ability.^2^ The range of donor sites that can match the pulp structure is limited.^3^^,^^4^ Moreover, the proper reconstructive modality must be appropriate for the size of the defect, the extent of damage, as well as patient individual factors such as occupation and postoperative functional requirements.

Options for reconstructing volar deficits include local homodigital or heterodigital flaps.^1^ Homodigital options include V-Y advancement flaps and Moberg flaps. These volar advancement flaps do not require microvascular anastomosis. Instead, the pedicle is left intact, and tissue that is proximally adjacent to the wound base is advanced distally to cover the defect. These are easily reproducible flaps that can also be sensitized and have no donor site morbidity. However, they can only cover smaller defects at the most distal thumb tip and are limited to wounds less than 1 to 2 cm.^1^^,^^5^ While heterodigital options such as the Littler neurovascular flap or the first dorsal metacarpal artery flap can reconstruct larger defects, they are a poor aesthetic match for the original thumb pulp soft tissue and can cause significant donor site morbidity.^3^^,^^5^

Injuries involving larger defects and loss of pulp or composite tissue may be better treated with a small free flap.^5^^,^^6^ Donor sites can be designed from various anatomical regions, including the forearm, groin, plantar or dorsal foot, and the pulp or lateral portion of the first or second toe. Of these options, free pulp and lateral toe flaps provide the best match for glabrous tissue coverage and result in better sensory recovery than other glabrous flaps, such as the medial plantar perforator artery flap or the free thenar flap.^4^

Recently, some case series have been published evaluating the utility of free flap reconstruction of thumb defects. Zheng et al^4^ reviewed 48 lateral great toe flap reconstructions, 20 of which involved thumb defects, and found a high flap survival rate with only one flap failure. They reported minimal donor site morbidity, good cosmesis, and satisfactory sensory recovery. Hung et al^6^ reported 4 cases of lateral great toe flap reconstructions, one of which involved thumb defects. All 4 flaps survived, and the authors reported good return to function and acceptable cosmesis.

### Surgical procedure

In our patient with a 7×3-cm open wound extending past the interphalangeal joint (Fig 1*a*), local flap options would have been insufficient in covering the soft tissue defect. Moreover, given the patient's background as a manual laborer, reconstruction of the defect with a robust, well-vascularized skin with a potential restoration of sensation was critical to a meaningful reconstruction. The lateral toe flap was ideal in this case, providing adequate tissue for coverage and having the appropriate tissue characteristics to facilitate functional recovery. After induction of general anesthesia, all devitalized tissue was debrided. A template of the wound was transferred to the lateral aspect of the left great toe, which included the area from the toe tip to the first web space (Fig 1*b*). A lazy-S incision centered over the dorsalis pedis artery was made. The vascular pedicle, consisting of the dorsal superficial vein of the foot and the dorsalis pedis artery, was identified, dissected, and isolated just distal to the ankle joint, following down to the great toe; the branch to the plantar arterial system was tied off. Mobilization of the flap was performed using loop magnification. The flap was then elevated from medial to lateral (Fig 1*c*). Unfortunately, intraoperatively, it was found that the digital nerve was not affiliated with the flap as expected, which may be an individual variation. A split-thickness skin graft from the thigh was used to cover the donor site defect. The recipient radial artery within the anatomical snuffbox and the recipient dorsal superficial vein were prepared under the microscope. Arterial anastomosis was performed in end-to-side fashion and the vein anastomosis in an end-to-end fashion with hand-sewn 9-0 nylon sutures. The nail bed was stented open, and the patient was placed in a volar wrist cock-up splint. The patient had a successful outcome and demonstrated satisfactory proprioceptive sensation over the volar aspect of the left thumb and a promising return to normal function (Fig 1*d*).

We present a report of successful salvage of an extensive volar thumb injury using a free lateral great toe flap. Reconstruction of extensive volar thumb defects must be carefully planned as local flap options, while popular, may be inappropriate for the scope of damage. The free lateral toe flap can provide sufficient and aesthetic coverage and facilitate functional recovery.
